# An Evidence-Based Approach to Teaching Obesity Management to Medical Students

**DOI:** 10.15766/mep_2374-8265.10662

**Published:** 2017-12-20

**Authors:** Magdalena Pasarica, Daniel Topping

**Affiliations:** 1 Associate Professor of Medicine, Medical Education Department, University of Central Florida College of Medicine; 2 Assistant Professor of Medicine, Medical Education Department, University of Central Florida College of Medicine

**Keywords:** Motivational Interviewing, Weight Loss, Obesity, Primary Care, Bariatric Surgery, Management, Lifestyle, Behavior Intervention

## Abstract

**Introduction:**

The need for education of future and current providers in evidence-based management of obesity and the release of new treatment guidelines prompted the development of a resource for use in the education of medical students and residents.

**Methods:**

A self-contained module was developed to provide an overview of recent guidelines for obesity management utilizing evidence-based medicine while debunking popular myths associated with available weight-loss strategies. The module was delivered over 15 months to six groups of learners (*N* = 180) and was continuously improved through feedback from content experts and the learners. After completion of the module, one subset of learners responded to a three-question survey using a 5-point Likert scale (1 = *strongly disagree,* 5 = *strongly agree*).

**Results:**

Formal evaluation of the module was completed by a subset of the learners (*N* = 32, 64% response rate). The majority agreed or strongly agreed with these survey statements: “Overall this module was valuable as an educational tool” (97%, *Mdn* = 4); “After completion of this module, I am confident of my knowledge on how to manage obesity in adult patients” (84%, *Mdn* = 4); and “It was easy to navigate the module” (94%, *Mdn* = 5).

**Discussion:**

This module could be implemented as is at other institutions that strive to educate medical students or residents on the most recent guidelines and evidence-based medicine regarding obesity management.

## Educational Objectives

By the end of this session, learners will be able to:

1. Provide clinically appropriate recommendations to patients with obesity regarding healthy lifestyle interventions, pharmacologic therapy, and weight-reduction surgery.
2. Use evidence-based medicine (EBM) to debunk popular myths associated with diet, activity, and behavior as they relate to the treatment of obesity.
3. Describe EBM recommendations that primary care physicians can use in clinical practice to manage patients with obesity.

## Introduction

The most recent statistics reveal that approximately one-third of US adults and 17% of children are obese.^1^ The National Academy of Sciences reports that obesity is a major contributor to the rate of chronic diseases in the US.^2^ Recent evidence shows that a significant number of adults are trying to either lose weight (43%) or maintain their current weight (23%); however, most physicians are not currently integrating available evidence-based medicine (EBM) guidelines and recommendations for advanced obesity management, attributing this to self-reported deficiencies in knowledge, skills, and confidence.^3^ In an effort to improve the effectiveness of obesity management, improve patient outcomes, and decrease health care–related costs, guidelines for advanced obesity management were released by the U.S. Preventive Services Task Force^4^; the American College of Cardiology, the American Heart Association, and The Obesity Society^5^; and the American Association of Clinical Endocrinologists and the American College of Endocrinology.^6^

The need for education of future providers in evidence-based obesity management and the release of new guidelines prompted the development of an educational resource focused on advanced obesity management. A needs assessment was performed at our institution with medical students, residents, and attending physicians. When learners are presented with 30–50 pages of guidelines, most of them are overwhelmed and miss the take-home points to be used in clinical practice. In addition to this, there exist multiple misconceptions related to the role of lifestyle management of chronic diseases. The learners also reported that they would like evidence-based resources to use with their patients. Therefore, we identified the need to prepare a resource in a concise format with clear and practical points from the most recent guidelines. The resource also integrates other pertinent national guidelines, including dietary guidelines for Americans,^7^ physical activity guidelines for Americans,^8^ National Institutes of Health recommendations for alcohol consumption,^9^ and the U.S. Department of Health and Human Services' guide to healthy sleep.^10^ Many common myths related to obesity management are discussed in concert with EBM data that debunk those myths.^11–35^ Several federal evidence-based resources recommended for use in patient care are presented.^9,22,36–43^ As the biggest deficiencies were identified in the area of lifestyle medicine for the management of obesity,^44^ the content is focused on the highly important and timely need for behavior change to achieve weight loss. Since much of the current focus in medical education is on independent learning, the resource was developed to be used as a self-learning module or, alternatively, as a PowerPoint in a conventional face-to-face setting. The audience for this resource is intended to be medical students (and possibly residents and physicians) seeking to understand the most recent guidelines and evidence related to obesity management and to improve their skills in the treatment of patients with obesity and subsequent health care outcomes. A basic understanding of obesity is the necessary prerequisite and is usually covered in the preclinical curriculum in medical schools and other health professional training programs.

Resources available in *MedEdPORTAL* briefly describe some of the obesity management guidelines published before 2012,^45^ 2013,^46^ and 2015^47^ with a less extensive presentation of evidence-based data for clinical practice. Other resources are primarily focused on the motivational interviewing and counseling part of obesity management,^48,49^ the role of active teaching in weight-loss programs, or interprofessional collaboration on obesity management.^50,51^ As a result, this module presents all the major current guidelines along with take-home messages, the most recent evidence-based data disproving management myths, and practical tips for use in the clinic utilizing information from an extensive review of the literature. The first version of the module was created by adapting a more comprehensive CME session delivered by Dr. Magdalena Pasarica to primary care physicians during the Florida Academy of Family Physicians Spring Forum (highly evaluated by the audience as very useful and well organized).

## Methods

The self-contained learning module (Appendix A) utilizes Articulate software (Articulate Global, 2017), which allows for viewing on any web browser that supports HTML and can be navigated on demand by the learner. It can be used by students as an independent-study self-learning module or as an in-class individual or collaborative exercise during didactic sessions and can be completed in 20–30 minutes. To view this web-based module, please open the zip file (Appendix A) and extract all of the files contained within to your desktop. To begin the module, simply open the presentation.html file.

The module contains up-to-date guidelines and evidence-based recommendations for obesity management as well as practical recommendations for an individual primary care provider in a busy outpatient environment (overview presented in the Table). The module begins with a presentation of the current obesity management guidelines and take-home messages integrating the various guidelines. The module then describes in a clinically relevant way the components of management: healthy lifestyle recommendations, comprehensive lifestyle interventions, pharmacological therapy, and bariatric surgery. We created all the diagrams and tables based on the evidence cited at the bottom of each slide in the presentation. Following this, popular myths are introduced and then debunked by evidence-based data. The module ends with practical recommendations for the busy primary care provider who will need to manage obesity using evidence-based recommendations in a 15-minute visit. After completion of the module, learners are required to answer two multiple-choice questions in order to test their knowledge. The learning objectives and the methods utilized in the resource are based on a literature review and guidance from primary care practitioners involved in undergraduate and graduate medical education.

**Table.** Overview of the Teaching Module Content

Section	Content Source
Guidelines for obesity management	• U.S. Preventive Services Task Force • AHA/ACC/TOS 2013 guidelines • AACE/ACE 2016 guidelines • Summary of guidelines
Healthy lifestyle recommendations	• Dietary Guidelines for Americans • Physical Activity Guidelines for Americans • Rethinking Drinking (NIH recommendations) • *Your Guide to Healthy Sleep* (U.S. Department of Health and Human Services recommendations)
Comprehensive intensive lifestyle intervention	• Components of comprehensive lifestyle intervention • Effectiveness—evidence-based data • Diet—EBM recommendations • Physical activity—EBM recommendations • Sleep—EBM recommendations • Behavior change—EBM recommendations
Myths debunked	• Diet myths debunked • Physical activity myths debunked • Behavior myths debunked
Pharmacological therapy	• Indications • Effectiveness • Tips for prescribers • Mechanism of action, dosage, side effects, safety, monitoring
Bariatric surgery	• Guidelines from AACE, TOS, ASMBS 2013, and ADA 2017 • Effectiveness and risks • Role of primary care physician
How to manage obesity in a 15-minute outpatient visit	• Guidelines • 5A approach • Motivational interviewing for lifestyle changes • SMARTER goal setting for lifestyle changes • Online tools for lifestyle changes

Abbreviations: AACE, American Association of Clinical Endocrinologists; ACC, American College of Cardiology; ACE, American College of Endocrinology; ADA, American Diabetes Association; AHA, American Heart Association; ASMBS, American Society for Metabolic and Bariatric Surgery; EBM, evidence-based medicine; NIH, National Institutes of Health; TOS, The Obesity Society.

This module was part of a mandatory didactic session in the internal/family medicine clerkship during the third year of a 4-year curriculum and the medicine boot camp (at the end of the fourth year for those students who matched for residency in family medicine and internal medicine). The session was delivered initially as an in-class exercise facilitated by an instructor with extensive knowledge of obesity management; then, it was converted into a self-learning module to enhance its use in different settings and institutions. This conversion was done with two purposes: to generalize use of the module by other institutions that may not have an expert for in-person teaching and to decompress didactic time in the clerkship.

Learner perception of the module effectiveness was evaluated using a survey (Appendix B) containing three questions (on a 5-point Likert scale; 1 = *strongly disagree,* 5 = *strongly agree*). Data were reported as median and interquartile range (IQR). The survey was adapted from a validated previously published survey.^52–54^ Suggestions for improvement were collected from free-text responses. The survey was filled out on paper by one group of fourth-year students (*N* = 32) after they had completed the self-learning module. The cohort of 32 students served as the validation group, with the learning module subsequently undergoing several iterations of development and revision. The updated version of the module is presented here.

## Results

Part or all of this module has been used in the training of medical students at our institution since May 2016 (*N* = 180 students in six groups). Its primary use has been as a part of an in-class exercise taught by Dr. Pasarica in the internal/family medicine clerkship didactic sessions and medicine boot camp. After each new block of clerkship during the academic year (every 12 weeks), we further refined the material using EBM and insights gained, including student feedback. We also refined the module by using feedback from primary care providers with expertise in the field.

Evaluation of the module (Figure) by fourth-year boot camp medicine students (*N* = 32 respondents, 64% response rate) revealed that the majority (97%) agreed or strongly agreed with the statement “Overall this module was valuable as an educational tool” (*Mdn* = 4, IQR = 4.00, 5.00). A similar majority of the students (84% and 94%, respectively) agreed or strongly agreed that “After completion of this module, I am confident of my knowledge on how to manage obesity in adult patients” (*Mdn* = 4, IQR = 4.00, 4.25), and that “It was easy to navigate the module” (*Mdn* = 5, IQR = 4.00, 5.00). The suggestions for improvement included “enlarging the tables for clarity” and “add quiz questions.” Based on these suggestions, the setting for the tables was modified for ease of reading. In addition, two multiple-choice questions were added.

**Figure.**
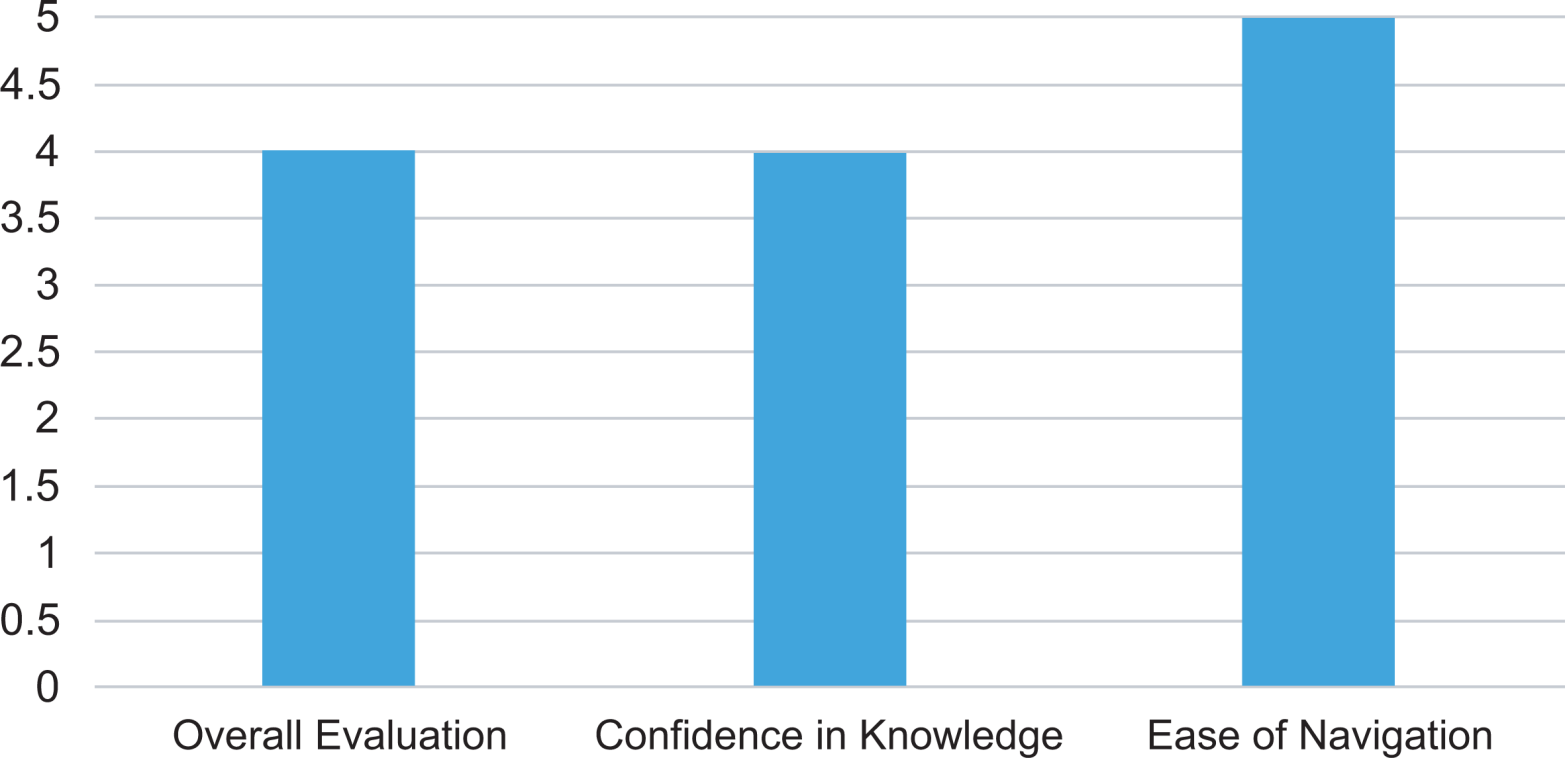
Student evaluation of the learning resource (median).

## Discussion

Current evidence reveals that most adult patients need and desire obesity management; however, providers are not adequately treating patients with obesity. There is a great deal of new and compelling evidence for advanced comprehensive obesity management. In light of this, our advanced obesity management module will be a useful resource for the education of medical students in the clinical years and/or primary care residents. The feedback we received from experts in the field, from primary care physicians, and from the medical students themselves has been positive, which supports the module's use in clinical undergraduate and graduate medical education. In less than 1 hour, learners are exposed to an extensive review of current guidelines and evidence related to obesity management in a clinically relevant way. The strengths of this resource include the multitude of evidence-based data presented, the need for education on the new guidelines, and the feedback from obesity experts. The module could be used in the future combined with a patient video of a clinical case or as a small-group collaborative exercise. This educational resource can be further enhanced by including a discussion of cases encountered in the learner's own clinical training experience on rotation. In addition to the survey presented here, a simulated exercise of managing the case of a patient with obesity, both before and after the session, could add to the impact of a learning session. If the resource is provided as an in-class exercise, facilitation by an instructor knowledgeable and experienced in the area of obesity medicine would be ideal for addressing gaps in knowledge and questions from students. If offered as a self-learning module, then no expertise is needed.

A limitation of this resource is that the current guidelines could be updated at any time; therefore, the instructor has to ensure that the resource is current prior to its delivery. As a result, we plan to periodically review and revise the resource and notify end users. The learners did not apply the module in a simulated patient scenario; if this had been the case, the students would have been able to apply the EBM recommendations rather than simply describe them, allowing them to achieve a higher level on Bloom's taxonomy of learning.

This module could be implemented as is at other institutions seeking to enhance the education of future health care providers on the most recent guidelines and evidence-based data related to obesity management. In addition, primary care physicians in practice could benefit from a refresher on the most recent evidence and guidelines.

## Appendices

A. Learning Module folder
B. Survey Instrument.docx All appendices are peer reviewed as integral parts of the Original Publication.
