# Supplementary material for: An Evidence-Based Approach to Teaching Obesity Management to Medical Students
Source: MedEdPORTAL. 2017 Dec 20;13:10662. doi: 10.15766/mep_2374-8265.10662 (PMC6338064; doi:10.15766/mep_2374-8265.10662)
Supplement: Supplementary file 1 — A. Learning Module folder B. Survey Instrument.docx [file mep-13-10662-s001.zip › B. Survey Instrument.docx]

**Appendix B:** Survey instrument for the assessment of learner’s perception of the module and suggestion for improvements

Regarding the module you just completed: An evidence based approach to obesity management

1. Rate your level of agreement with the following statements:
2. Overall this module was valuable as an educational tool

- Strongly agree
- Agree
- Neutral
- Disagree
- Strongly disagree

1. After completion of this module, I am confident of my knowledge of how to manage obesity in adult patients

- Strongly agree
- Agree
- Neutral
- Disagree
- Strongly disagree

1. It was easy to navigate the module

- Strongly agree
- Agree
- Neutral
- Disagree
- Strongly disagree

1. Do you have any feedback for the improvement of the module? We appreciate your comments

_______________________________________________________________________________________________________________________________________________________________________________________________________________________________________________________________________________________
