# Supplementary material for: An Evidence-Based Approach to Teaching Obesity Management to Medical Students
Source: MedEdPORTAL. 2017 Dec 20;13:10662. doi: 10.15766/mep_2374-8265.10662 (PMC6338064; doi:10.15766/mep_2374-8265.10662)
Supplement: Supplementary file 1 — A. Learning Module folder B. Survey Instrument.docx [file mep-13-10662-s001.zip › A. Learning Module folder/presentation_html5.html]

An Evidence Based Approach to Obesity Management


An Evidence Based Approach to Obesity Management

Drag up for fullscreen
