# Supplementary figures and images for: An Evidence-Based Approach to Teaching Obesity Management to Medical Students
Source: MedEdPORTAL. 2017 Dec 20;13:10662. doi: 10.15766/mep_2374-8265.10662 (PMC6338064; doi:10.15766/mep_2374-8265.10662)

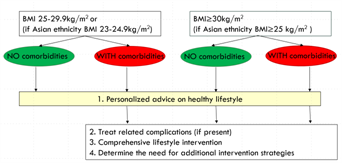

Supplement: Supplementary file 1 — A. Learning Module folder B. Survey Instrument.docx [file mep-13-10662-s001.zip › A. Learning Module folder/mobile/65486RyrL7V_None_342163_DX684_DY684_CX342_CY163.png]

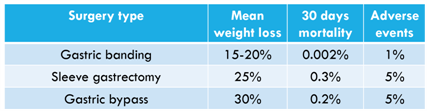

Supplement: Supplementary file 1 — A. Learning Module folder B. Survey Instrument.docx [file mep-13-10662-s001.zip › A. Learning Module folder/mobile/67zW0XIACV6_None_427111_80_DX854_DY854_CX427_CY111.png]

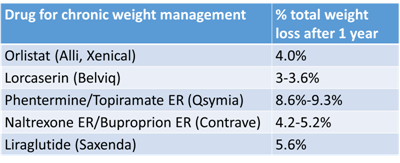

Supplement: Supplementary file 1 — A. Learning Module folder B. Survey Instrument.docx [file mep-13-10662-s001.zip › A. Learning Module folder/mobile/683GD5j5ocU_None_400163_DX800_DY800_CX400_CY163.png]

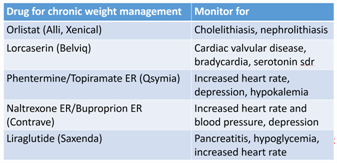

Supplement: Supplementary file 1 — A. Learning Module folder B. Survey Instrument.docx [file mep-13-10662-s001.zip › A. Learning Module folder/mobile/6ChqMYEDTZA_None_337163_DX674_DY674_CX337_CY163.png]

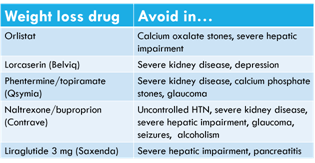

Supplement: Supplementary file 1 — A. Learning Module folder B. Survey Instrument.docx [file mep-13-10662-s001.zip › A. Learning Module folder/mobile/6f1jZuqYI3z_None_315163_DX630_DY630_CX315_CY163.png]

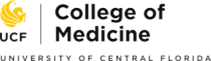

Supplement: Supplementary file 1 — A. Learning Module folder B. Survey Instrument.docx [file mep-13-10662-s001.zip › A. Learning Module folder/mobile/6GyGJqQb87x.png]

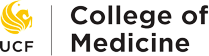

Supplement: Supplementary file 1 — A. Learning Module folder B. Survey Instrument.docx [file mep-13-10662-s001.zip › A. Learning Module folder/mobile/6Qgk7g4w30D_sl296shp4_DX314_DY314_CX209_CY56.png]

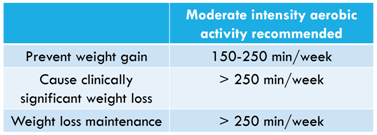

Supplement: Supplementary file 1 — A. Learning Module folder B. Survey Instrument.docx [file mep-13-10662-s001.zip › A. Learning Module folder/mobile/6S3KU0V6fI9_None_377135_80_DX754_DY754_CX377_CY135.png]

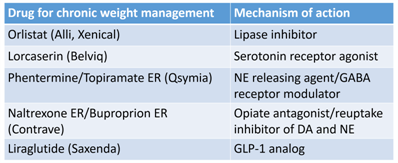

Supplement: Supplementary file 1 — A. Learning Module folder B. Survey Instrument.docx [file mep-13-10662-s001.zip › A. Learning Module folder/mobile/6UJsIHSCdhB_None_398163_DX796_DY796_CX398_CY163.png]

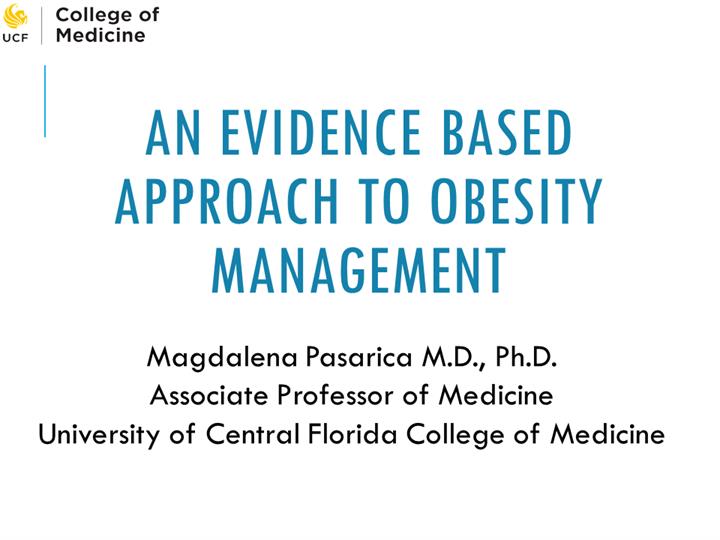

Supplement: Supplementary file 1 — A. Learning Module folder B. Survey Instrument.docx [file mep-13-10662-s001.zip › A. Learning Module folder/presentation_content/thumbnail.jpg]
